# Role of the K^+^-Cl^–^ Cotransporter KCC2a Isoform in Mammalian Respiration at Birth

**DOI:** 10.1523/ENEURO.0264-18.2018

**Published:** 2018-10-23

**Authors:** Christophe J. Dubois, Laura Cardoit, Veronika Schwarz, Marika Markkanen, Matti S. Airaksinen, Pavel Uvarov, John Simmers, Muriel Thoby-Brisson

**Affiliations:** 1 Institut de Neurosciences Cognitives et Intégratives D’Aquitaine, CNRS UMR 5287, Université de Bordeaux, Bordeaux 33076, France; 2 Department of Anatomy, Faculty of Medicine, University of Helsinki, Helsinki Finland

**Keywords:** Apnea, breathing, KCC2a, neural network, rhythmogenesis

## Abstract

In central respiratory circuitry, synaptic excitation is responsible for synchronizing neuronal activity in the different respiratory rhythm phases, whereas chloride-mediated inhibition is important for shaping the respiratory pattern itself. The potassium chloride cotransporter KCC2, which serves to maintain low intraneuronal Cl^–^ concentration and thus render chloride-mediated synaptic signaling inhibitory, exists in two isoforms, KCC2a and KCC2b. KCC2 is essential for functional breathing motor control at birth, but the specific contribution of the KCC2a isoform remains unknown. Here, to address this issue, we investigated the respiratory phenotype of mice deficient for KCC2a. *In vivo* plethysmographic recordings revealed that KCC2a-deficient pups at P0 transiently express an abnormally low breathing rate and a high occurrence of apneas. Immunostainings confirmed that KCC2a is normally expressed in the brainstem neuronal groups involved in breathing (pre-Bötzinger complex, parafacial respiratory group, hypoglossus nucleus) and is absent in these regions in the KCC2a^–/–^ mutant. However, in variously reduced *in vitro* medullary preparations, spontaneous rhythmic respiratory activity is similar to that expressed in wild-type preparations, as is hypoglossal motor output, and no respiratory pauses are detected, suggesting that the rhythm-generating networks are not intrinsically affected in mutants at P0. In contrast, inhibitory neuromodulatory influences exerted by the pons on respiratory rhythmogenesis are stronger in the mutant, thereby explaining the breathing anomalies observed *in vivo*. Thus, our results indicate that the KCC2a isoform is important for establishing proper breathing behavior at the time of birth, but by acting at sites that are extrinsic to the central respiratory networks themselves.

## Significance Statement

The expression of the neuronal specific potassium chloride cotransporter KCC2 is necessary for the generation of respiratory activity at birth, but the specific role of the KCC2a isoform in this process remains unknown. By examining *in vivo* and *in vitro* the breathing motor output of newborn KCC2a^–/–^ mice, we observed a lower breathing frequency and a high occurrence of apneas at birth. These anomalies, expressed mainly at P0 (day of birth), do not arise from changes in the brainstem respiratory rhythm-generating circuits, but appear to result from abnormally strong inhibitory pontine neuromodulatory influences that target these networks. Our results provide evidence for a transient but important role of the KCC2a isoform in the proper development of the central respiratory command.

## Introduction

The development of functional neuronal circuits requires the establishment of appropriate excitatory and inhibitory synaptic signaling between interconnected neurons and circuits. One important function with vital physiologic relevance is breathing, which is controlled by interacting neuronal assemblages located in the brainstem. In the respiratory rhythm-generating networks, synaptic excitation is required for activity synchronization within synergistic neuronal pools, whereas inhibitory synaptic transmission is mostly implicated in pattern formation, the regulation of neuronal excitability, and the activation of intrinsic membrane properties (Shao and Feldman, 1997; Brockhaus and Ballanyi, 1998; Ren and Greer, 2006; Morgado-Valle et al., 2010; Janczewski et al., 2013; Smith et al., 2013; Chapuis et al., 2014; Richter and Smith, 2014; Sherman et al., 2015; Marchenko et al., 2016; Ramirez et al., 2016; Baertsch et al., 2018).

In the central nervous system (CNS), inhibitory synaptic neurotransmission relies on chloride ion movements through transmembrane channels, the direction of which depends directly on chloride ion gradients. Neuronal chloride homeostasis is mainly ensured by two types of cation-chloride cotransporters, the Na^+^-K^+^-2Cl^–^ cotransporter NKCC1 that participates in accumulating chloride in the neuronal intracellular compartment (Sun and Murali, 1999) and the K^+^-Cl^–^ transporter KCC2, which extrudes chloride from neurons (Payne et al., 1996; Rivera et al., 1999). The chloride ion gradient results from the difference between intra- and extracellular Cl^–^ concentrations. Thus, the excitatory or inhibitory nature of chloride-mediated signaling depends on a fine balance between the expression and functional state of the Cl^–^ cotransporters NKCC1 and KCC2 in the cellular membrane (Rivera et al., 1999), with anomalies in this relationship having important pathologic consequences (Ben-Ari et al., 2012; Kaila et al., 2014).

KCC2 is abundantly expressed in the vast majority of mammalian central neurons, with a very low expression occurring in neurons of the peripheral nervous system and in non-neuronal cell types such as glia. Two isoforms differing by their N-terminal sequences have been described for KCC2 (Uvarov et al., 2007, 2009; Markkanen et al., 2014). Both isoforms, KCC2a and KCC2b, are expressed in several regions of the CNS, including the hindbrain (Uvarov et al., 2009; Markkanen et al., 2014). Transgenic mice deficient for the two KCC2 subtypes die at birth because of severe deficits in motor control, including that KCC2 is responsible for breathing (Hübner et al., 2001). In contrast, knockout mice for KCC2a alone survive until adulthood without exhibiting any obvious major deficits, which is possibly due to a compensatory process involving the still present and related KCC2b isoform (Markkanen et al., 2014). On the other hand, mice deficient solely for the KCC2b isoform survive for 2 to 3 weeks (Woo et al., 2002), thereby suggesting a transient but nevertheless important role for KCC2a in early postnatal respiratory function. To further address this possibility, we characterized the respiratory phenotype caused by a KCC2a mutation at birth, and by using variously reduced types of isolated brainstem preparations, we explored the impact of a KCC2a deletion on the rhythmogenic capability of respiratory circuitry *in vitro.* We show that despite the finding that KCC2a mutants survive and subsequently appear to develop normally, they transiently exhibit respiratory anomalies at P0, confirming that the KCC2a isoform normally contributes to proper respiratory network function at birth. However, our data indicate that KCC2a’s regulatory role may be accomplished in pontine assemblages upstream from the medullary rhythm-generating networks, rather than within neurons of the respiratory motor circuits themselves.

## Materials and Methods

All animal procedures were performed in accordance with the University of Bordeaux animal care committee’s regulations. Mice were housed under standard conditions with a 12/12-h dark/light cycle and food and water provided *ad libitum*.

### Animals

The *KCC2a* mouse strain (background strain C57BL/6Hsd; Markkanen et al., 2014) that specifically disrupts the KCC2a isoform was maintained and bred in our animal facilities. Because homozygous animals survive and breed normally, they were intercrossed to generate litters containing exclusively KCC2a^–/–^ embryos. Wild-type control animals were obtained from the same mouse line (intercrossed male and female KCC2a^+/+^). All experiments were performed at postnatal day 0 (P0, day of delivery), except for plethysmographic recordings that were performed from P0 up to P5.

### Plethysmographic recordings

Breathing parameters were measured from unanesthetized and unrestrained pups using a whole-body plethysmograph. P0–P5 newborn mice were placed inside a 50 ml chamber for recording sessions lasting 5 min. The chamber was placed under a heating lamp to avoid cooling of experimental animals and to maintain a constant temperature throughout the entire recording period. The chamber was not supplied with a continuous flow of fresh air (as is commonly used to avoid CO_2_ accumulation), since this procedure was found to introduce artifactual noise that disrupted readout and measurement of the low-amplitude respiratory movements produced by the small P0 animals. Under this condition, however, no sign of asphyxia was observed after 5 min of recording, indicating that the chamber volume was sufficiently large relative to the quantity of CO_2_ produced by such young pups. The chamber was connected to a data acquisition system (Emka Technologies) that measured flow and pressure changes within the chamber using Iox2 software. Data were sampled at 1 kHz. Recordings were analyzed offline using PClamp10 software (Molecular Devices). Breathing frequency was calculated on a breath-to-breath basis. Interruptions in the breathing rhythm were considered as apneas when at least 1.5 ongoing cycles were skipped. Because of the small size of animals at P0 and their very limited respiratory volumes, to avoid obtaining erroneous measurements we did not analyze other classically tested breathing parameters such as tidal volume, ventilation, and the durations of inspiration and expiration.

### *In vitro* preparations

Three types of *in vitro* preparations at various stages of isolation were used in the present study: pontomedullary preparations containing the neuronal circuits of the pons and the medulla; isolated medullary preparations (brainstem) that contain the two main respiratory networks, the pFRG and the pre-Bötzinger complex (preBötC); and transverse brainstem slices containing the preBötC network alone. All preparations were dissected in an artificial CSF (aCSF) solution composed of (in mm): 120 NaCl, 8 KCl, 1.26 CaCl_2_, 1.5 MgCl_2_, 21 NaHCO_3_, 0.58 NaH_2_PO_4_, and 30 glucose, pH 7.4, maintained at 4°C and continuously bubbled with a mixture of 95% O_2_ and 5% CO_2_. Experimental newborn animals were decerebrated and decapitated. Pontomedullary preparations were isolated from the heads by a rostral section performed at the junction between the rhombencephalon and mesencephalon and a caudal section made below the sixth cervical roots. Surrounding tissue was carefully removed to keep intact ventral cervical roots required for electrophysiological recordings. For medullary preparations, the pons was removed by an additional transverse section performed at the rostral border of the facial nucleus to eliminate the neuromodulatory influences that rostralmost structures exert on the respiratory networks (Abdala et al., 2009; Dutschmann and Dick, 2012; Smith et al., 2013; Bautista and Dutschmann, 2014). Transverse brainstem slices were further obtained by serially sectioning in the rostral-to-caudal direction isolated hindbrains mounted in a low-melting-point agar block using a vibratome (Leica VS 1000). A 450-µm-thick slice isolating the preBötC was obtained at the axial level that was ∼300 µm more caudal to the posterior limit of the facial nucleus. Other anatomic landmarks were also used, such as the opened fourth ventricule, the inferior olive, nucleus ambiguus, and hypoglossal nucleus, all of which should be detectable in the slice (Thoby-Brisson et al., 2005; Ruangkittisakul et al., 2011). All *in vitro* preparations were then positioned ventral (pontomedullary and medullary preparations) or rostral (transverse slice) side up in the recording chamber and continuously bathed in circulating oxygenated aCSF (as detailed above) at 30°C.

### Electrophysiological recordings of neuronal activity

With pontomedullary and medullary preparations, the activity of the phrenic nerve ventral root (C4) or that of the hypoglossal root (XII) was recorded using a suction electrode fabricated from glass tubing broken at the tip to match the diameter of the root in question. In transverse brainstem slices, preBötC population activity was recorded using a glass macropipette positioned at the surface of the slice in the region containing the preBötC respiratory network (in the proximity of the nucleus ambiguus). The recording pipette was filled with aCSF and connected through a silver wire to a high-gain amplifier (AM Systems). Signals were filtered (bandwidth 3 Hz to 3 kHz), rectified, integrated (time constant 100 ms; Neurolog, Digitimer), recorded, and analyzed offline on a computer through a Digidata 1440 interface and PClamp10 software (Molecular Devices). Fictive respiratory frequency was measured over periods of 2–3 min. Statistical differences in frequencies were estimated using the Mann–Whitney rank sum test. Differences were considered significant at *p* < 0.05. An irregularity of respiratory periodicity was determined for each cycle by applying the following formula for consecutive cycle duration values: IS*n* = 100 * ABS[(P*n* – P*n*–1)/P*n*–1], with IS being the irregularity score of the *n*th cycle, P*n* its period, P*n* – 1 the period of the preceding cycle, and ABS the absolute period value (Barthe and Clarac, 1997). Thus, the lower the IS value, the more regular was the ongoing respiratory rhythm.

### Pharmacology

Pharmacological agents were obtained from Sigma and dissolved in oxygenated aCSF for bath-application lasting 15–30 min at a final concentration of 1 µm for substance P (SP) to activate NK1R receptors and 50 µm for yohimbine to block α2-AR. The drugs’ effects on rhythm generation were assessed at the end of the exposure period. Burst cycle frequency values are given as means ± SEM. Statistical significance was assessed by Student’s *t* test or Mann–Whitney when appropriate. Mean values were considered as statistically different when *p* < 0.05.

### Immunostaining

For immunostaining procedures, brainstem preparations were placed in 4% paraformaldehyde for 2–3 h for tissue fixation. Transverse or sagittal frozen sections were obtained by placing brainstems overnight in a 20% sucrose-PBS solution for cryoprotection, then embedding them in a block of Tissue Tek (Leica) and sectioning at 30 µm using a cryostat (Leica). To limit nonspecific labeling, preparations were incubated for 90 min in a solution of PBS containing 0.3% Triton X-100 and 1% BSA. Primary antibodies were then applied overnight at room temperature and under slight agitation. As primary antibodies, we used goat anti-ChAT (1:100; Millipore) as a marker for cholinergic motoneurons, rabbit anti-KCC2a (1:1000; Uvarov et al., 2009), chicken anti-KCC2b (1/300, Uvarov et al., 2009), or rabbit anti-NK1R (1:10,000; Sigma) as markers for KCC2a, KCC2b, and NK1R, respectively. The specificity of the anti-KCC2a and -KCC2b antibodies employed here has been described previously in detail (Uvarov et al., 2009; Markkanen et al., 2014, 2017). Then, after several washes, preparations were incubated with secondary antibodies for 90 min at room temperature. These secondary antibodies (1:500; Invitrogen) were an Alexa Fluor 568–conjugated donkey anti-rabbit antibody, an Alexa Fluor 488–conjugated donkey anti-chicken antibody, and an Alexa Fluor 488–conjugated donkey anti-goat antibody. Stained slices were coverslipped and mounted in Vectashield Hard Set medium (Eurobio) and kept in the dark until imaging using an epifluorescence microscope (Olympus).

## Results

### Breathing of KCC2a^–/–^ mice during the first postnatal week

**Figure 1.**
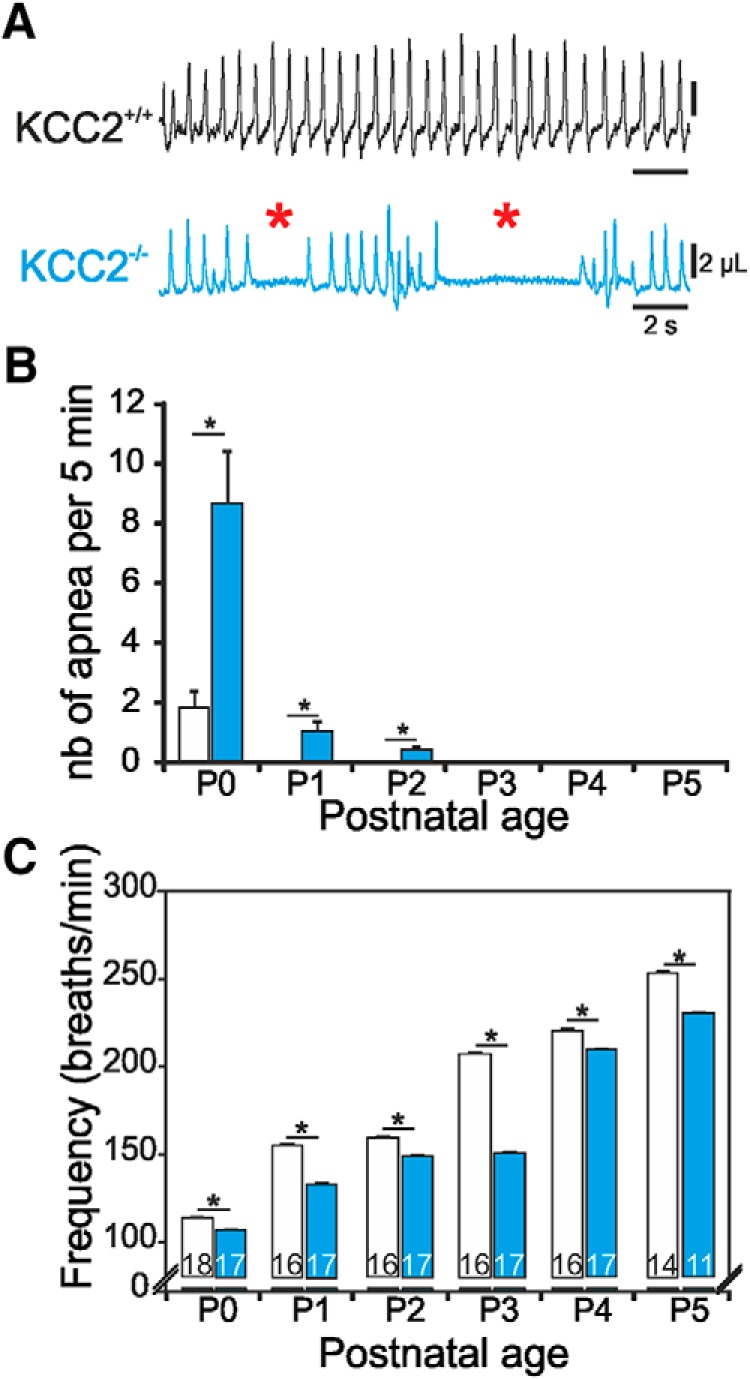
KCC2a^–/–^ pups at P0 exhibit an abnormally low breathing rate and high occurrence of apneas. ***A***, Whole-body plethysmographic recordings of breathing behavior of a wild-type (KCC2a^+/+^, black trace) and a mutant (KCC2a^–/–^, blue trace) newborn mouse just after birth. Red asterisks indicate recurrent apneic episodes that were absent in equivalent recordings from wild-type animals. Note also the slower breathing rate of the mutant outside apneic-like pauses. ***B***, ***C***, Quantification of the mean number of apneas per 5-min recordings (***B***) and mean respiratory frequency (***C***) measured in wild-type (unfilled bars) and KCC2a^–/–^ (blue bars) pups *in vivo* over the first 5 postnatal days. Numbers on bars indicate the number of animals analyzed. Black asterisks indicate statistical differences (*p* < 0.05). Whereas wild-type pups breathe regularly and continuously at P0, KCC2a mutant pups breathe relatively slowly and exhibit a high degree of respiratory pauses.

Using whole-body plethysmographic recordings, we compared the breathing behavior of KCC2a^+/+^ (*n* = 18) and KCC2a^–/–^ (*n* = 17) newborn mice during the 6 d following birth (P0–P5). Respiratory cycle frequencies and the number and duration of apneas were measured during 5-min recording sessions, with an individual apnea being defined as a respiratory cycle exceeding 150% of the mean control cycle period. At P0, frequent apneas were observed in 16/17 KCC2a mutant animals examined, with a mean of 8.7 apneic episodes occurring per monitoring session (Fig. 1*A*, *B*). The mean duration of apneas was 3.04 ± 0.64 s, corresponding to 8.8% of the total recording time spent in the apneic respiratory state. This proportion of apneas decreased significantly to 0.3% at P1 and 0.15% at P2, until respiratory pauses were no longer detected at subsequent older stages (P3–P5). In contrast, only 50% of the wild-type P0 pups (9/18) expressed apneas, with a mean occurrence of 1.7 per 5-min test period, and a mean duration of 1.96 ± 0.54 s corresponding to 1.1% of the total recording time. No apneas were evident from P1 onward in wild-type pups (Fig. 1*B*).

Measurements of breathing frequency during non-apneic periods (i.e., outside pauses) revealed significant differences between the mean rate values of the KCC2a mutant versus wild-type animals. KCC2a^–/–^ pups breathed at a significantly slower frequency at all developmental stages tested (P0 to P5; Mann–Whitney test, *p* < 0.001 for each stage; Fig. 1*A*, *C*). For example, at P0, the respiratory frequency was 113 ± 0.5 breaths/min in KCC2a^+/+^ versus 106 ± 0.6 breaths/min in KCC2a^–/–^ animals. Although the KCC2a mutants then showed a developmental increase in breathing frequency over the P0–P5 period, as in the control population (Fig. 1*C*), the respiratory rate at each developmental stage remained significantly lower than the corresponding value in KCC2a^+/+^ animals. These findings thus indicate that newborn mice deficient for KCC2a exhibit a slower breathing frequency and a higher rate of apneas than their control counterparts, thereby suggesting that the proper expression of KCC2a at the time of birth is required for the generation of normal breathing behavior. Because these breathing anomalies were most evident at P0, we then focused on this specific developmental stage to explore the underlying central causes using *in vitro* preparations.

### Expression of KCC2a in brainstem respiratory nuclei of newborn mice

**Figure 2.**
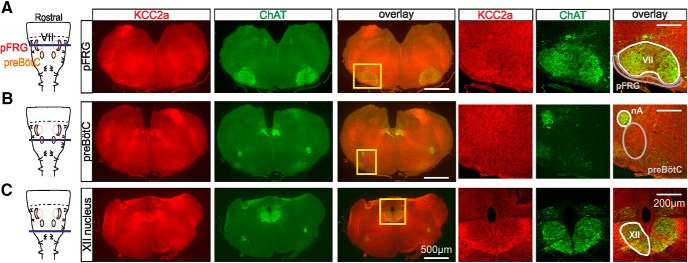
The KCC2a cotransporter is expressed in brainstem regions containing neuronal populations involved in respiration. ***A–C***, Immunostainings against KCC2a (red) and ChAT (green) in transverse, 30-µm-thick brainstem slices obtained from a P0 KCC2a^+/+^ animal at the axial level of the parafacial respiratory group (***A***), the preBötC (***B***), and the hypoglossus nucleus (***C***). Left: Schematic representations of isolated medullary preparations containing the pFRG (red shading) and the preBötC (orange shading). The violet horizontal line in each schematic indicates the rostro-caudal level corresponding to the images at right. The yellow rectangles in the middle overlay panels delimit the areas encompassing the pFRG, the preBötC, and the hypoglossus nucleus and are presented at a higher magnification in the three righthand panels in each case. Note that KCC2a is strongly expressed in these three neuronal groups that are all critically involved in generating respiration. VII, facial motor nucleus; nA, nucleus ambiguus; pFRG, parafacial respiratory group; XII, hypoglossus nucleus.

To further investigate the functional consequences of a KCC2a-targeted mutation on respiration, we first assessed the anatomic expression of KCC2a at P0 in brainstem areas where the respiratory motor rhythm–generating networks are known to reside. To this end, immunostaining directed against KCC2a was performed using a homemade specific antibody (Uvarov et al., 2009; Markkanen et al., 2014) in conjunction with a ChAT antibody to locate brainstem motoneuronal groups (Ericson et al., 1992) that neighbor the respiratory networks. These adjacent groups include the nucleus ambiguus (nA) and the facial motor nucleus landmarking the preBötC region and the pFRG, respectively, and the hypoglossal nucleus controlling the upper airway musculature. First, at the axial level of the pFRG, KCC2a-positive staining was found in a large region covering the ventralmost two-thirds of each slice. (Fig. 2*A*, three left panels). At a higher magnification, a significant staining was visible in the area lying ventral to the facial nucleus where the pFRG network is located (Fig. 2*A*, three right panels). It is noteworthy that KCC2a was heterogeneously expressed in the facial motor nucleus, with a more pronounced labeling occurring in the most lateral subnucleus. Second, at the axial level of the preBötC, KCC2a was again expressed in a large proportion of the tissue, including the region encompassing the preBötC area (Fig. 2*B*). Third, at a more caudal level where closure of the fourth ventricule is starting, strong KCC2a staining was found in the region where the hypoglossal nuclei are located (Fig. 2*C*), as well as in their entire rostrocaudal extensions. Note that, as previously reported (Markkanen et al., 2014, 2017), the pattern of KCC2a labeling is rather diffuse because KCC2a immunoreactivity is generally detected mainly in distal dendrites and is barely visible in neuronal somata and proximal dendrites. Altogether, therefore, these immunostaining data indicate that KCC2a is strongly expressed at P0 in hindbrain regions containing the preBötC and pFRG, the two main respiratory oscillators, and in motor nuclei also involved in respiratory function. Accordingly, the breathing anomalies observed in KCC2a mutant newborns might indeed be directly due to a dysfunction of the neuronal circuits that orchestrate the central breathing command.

### PreBötC network activity *in vitro* is not affected at birth by the KCC2a^–/–^ mutation

**Figure 3.**
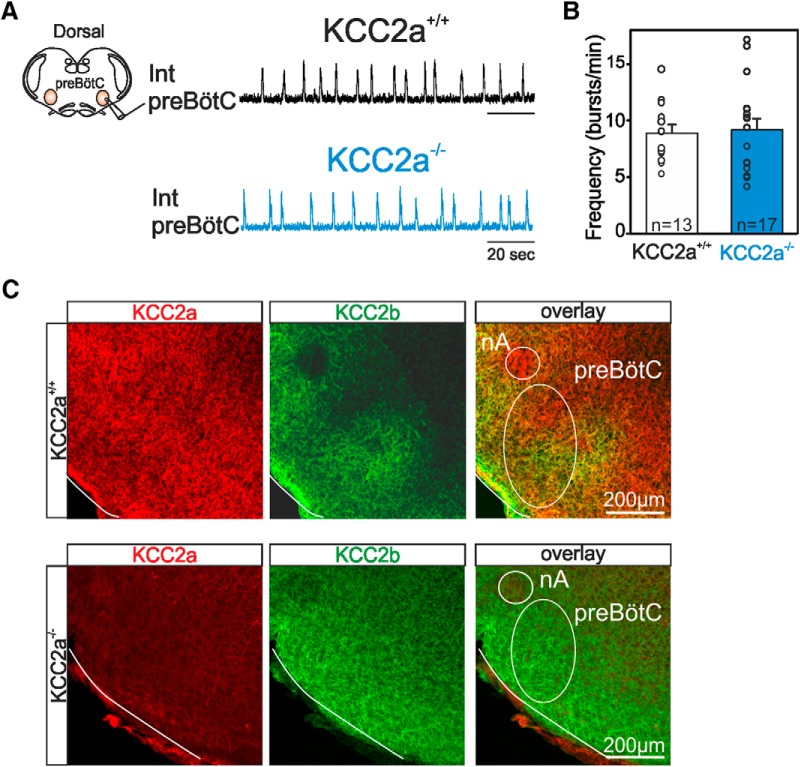
Rhythmogenesis by the isolated preBötC network is unaltered in KCC2a^–/–^ mutants at P0. ***A***, Left: Schematic representation of a transverse brainstem slice preparation isolating the preBötC network (in orange) with the recording macroelectrode positioned on the slice surface to monitor spontaneous preBötC network activity. Right: Integrated recording traces of preBötC activity in a preparation obtained from a KCC2a^+/+^ (black trace) and a KCC2a^–/–^ (blue trace) newborn mouse at P0. Note the absence of fictive inspiratory pauses in the mutant. ***B***, Histograms representing individual mean frequency values (open circles) and the corresponding mean (± SEM) values for preBötC network activity measured in KCC2a^+/+^ (*n* = 13; unfilled bar) and KCC2a^–/–^ (*n* = 17; blue bar) preparations. The inspiratory burst frequencies expressed by the preBötC network are comparable in the two genotypes. ***C***, Immunostainings against KCC2a (red) and KCC2b (green) in transverse brainstem slices obtained from a P0 KCC2a^+/+^ (top) and KCC2a^–/–^ (bottom) animal at the axial level of the preBötC. Note the coexpression of both isoforms in the WT and the complete absence of KCC2a in the mutant (the slight background labeling is substantially below the positive labeling obtained in preparations from wild-type animals and thus does not correspond to any significant immunoreactivity).

Inspiration, the major component of the respiratory rhythm, is primarily generated by rhythmogenic circuitry in the preBötC (Smith et al., 1991). To test whether the frequency alteration observed in KCC2a-deficient mutants *in vivo* could be caused by a specific dysfunction of the preBötC, we used transverse brainstem *in vitro* slice preparations that completely isolate this network (see Methods). Spontaneous preBötC inspiratory activity was recorded from 13 such preparations obtained from KCC2a^+/+^ pups and 17 preparations obtained from KCC2a^–/–^ pups. Sample recordings from each genotype are illustrated in Fig. 3*A*. The mean rhythm frequency values for the KCC2a^+/+^ preparations (8.9 ± 0.7 bursts/min) and KCC2a^–/–^ preparations (9.2 ± 0.9 bursts/min) were not statistically different (*p* = 0.8; Fig. 3*B*). Moreover, no sign of interruptions corresponding to respiratory pauses were detected throughout the entire recording sessions for all preparations tested. Therefore, these findings indicate that preBötC network function remains unaffected in the mutant and that the breathing anomalies observed *in vivo* do not originate from deficits in preBötC operation alone.

A mechanism allowing the preBötC network to appear to function properly in the mutant could derive from a functional overlap between the two KCC2 isoforms, whereby the lack of one isoform (here KCC2a) is compensated for by the other (KCC2b), as previously proposed in other brain structures (Markkanen et al., 2014). To test the basis for this possibility, we verified the expression of KCC2b in the region encompassing the preBötC. As shown in Fig. 3*C* (upper panels), both KCC2a and KCC2b were coexpressed in the preBötC region of wild-type animals. In the mutant, as expected, KCC2a was absent in the preBötC (Fig. 3, lower panels) as well as in all inspected brainstem regions (data not shown), in contrast to KCC2b, whose continued presence indicated that this isoform was indeed in a position to potentially compensate for the lack of KCC2a.

### Combined pFRG and preBötC network activity *in vitro* is not affected at birth by the KCC2a^–/–^ mutation

**Figure 4.**
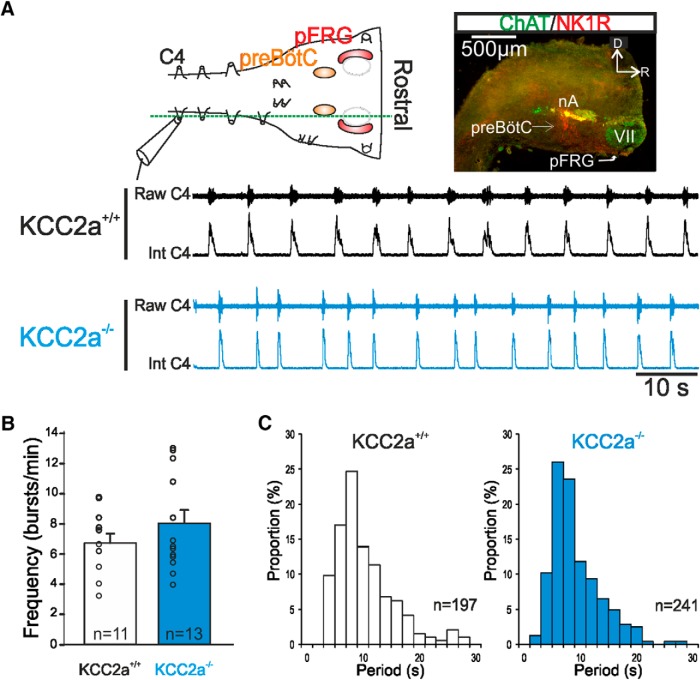
Respiratory rhythmogenesis by the isolated preBötC and pFRG networks is unaltered in KCC2a^–/–^ mutants at P0. ***A***, Top left: Schematic representation of an isolated medullary preparation containing the pFRG (red shading) and preBötC (orange shading) networks with a suction electrode placed on a phrenic rootlet (C4) to record spontaneous rhythmic respiratory activity. The dashed green line indicates the sectioning level used to obtain the image at top right. Top right: Sagittal slice of a hindbrain preparation with the localization of motoneuronal groups (from ChAT immunostaining in green) demarking the rostral margin of the preparation. Bottom: Recordings of raw (top) and integrated (bottom) C4 activity in preparations obtained from a wild-type (upper black traces) and a KCC2a^–/–^ (lower blue traces) newborn mouse. Note the absence of fictive apneas in the mutant preparation. ***B***, Histograms showing the overall mean (± SEM) cycle frequency values measured from 11 KCC2a^+/+^ (unfilled bar) and 13 KCC2a^–/–^ (blue bar) isolated medullary preparations, together with mean values for individual preparations (open circles) obtained over a 3-min recording period. The fictive respiratory rhythm rate was not significantly different in KCC2a^–/–^ mutant preparations compared to wild-type preparations at P0. ***C***, Histograms of cycle period distributions for 197 respiratory bursts measured in a KCC2a^+/+^ preparation (left, unfilled bars) and 241 respiratory bursts in a KCC2a^–/–^ preparation (right, blue bars). No significant differences in the interburst interval distributions are evident, further indicating the similarity between that the rhythmic respiratory activities in isolated medullary preparations from the two genotypes.

It is now well established that the preBötC network is functionally interconnected to the pFRG to form a dual oscillator system for the generation of breathing (Mellen et al., 2003; Feldman and Del Negro, 2006; Thoby-Brisson et al., 2009; Bochorishvili et al., 2012). To test whether the respiratory anomalies observed in KCC2a^–/–^ newborn pups arose from deficits in the preBötC’s neighboring pFRG network and/or in their strong synaptic interactions (see Discussion), we recorded spontaneously produced respiration-related activity in isolated medullary preparations that preserve both respiratory networks (Fig. 4*A*, top). In such preparations obtained from KCC2a^+/+^ animals, rhythmic inspiratory motor bursts recorded from a phrenic nerve root (C4) were generated at a mean frequency of 6.7 ± 0.6 bursts/min (*n* = 11; Fig. 4*A*, top, and 4*B*). In equivalent recordings of fictive respiratory activity in isolated preparations from KCC2a^–/–^ newborns (*n* = 13), the mean frequency of spontaneous C4 bursts was 7.9 ± 0.8 bursts/min, a slightly higher value that was not statistically different from that of control preparations (*t*-test, *p* = 0.3; Fig. 4*A*, bottom, and 4*B*). Moreover, no sign of any intermittent longer periods between consecutive rhythm cycles that would correspond to the apneas seen *in vivo* was evident in mutant *in vitro* preparations. Further confirmation of the complete absence of fictive respiratory pauses can be seen in the group data histograms of Fig. 4*C*, where period distributions for both KCC2a^+/+^ (at left) and mutant preparations (at right) are plotted: the two plots display a comparable repartition of rhythm cycle periods, without any obvious longer periods that would have indicated the occurrence of apneic-related events in the mutant preparations. We also calculated the irregularity score (IS, see Methods) of the rhythmic respiratory activity recorded in medullary preparations, in reasoning that the occurrence of any fictive apneas would affect the regularity (i.e., by increasing IS) of the ongoing respiratory rhythm. However, the mean IS value for mutant KCC2a^–/–^ preparations (50.0 ± 4.4, *n* = 7) was not statistically different from that of control KCC2a^+/+^ preparations (40.7 ± 4.4, *n* = 9; Mann–Whitney test, *p* = 0.6), indicating that the fictive respiratory rhythm was as regular in the mutant as in the wild type, and was not subjected to any significant activity interruptions. Taken together, therefore, these results demonstrate that the neural signaling for the apneas and slower breathing rate occurring in KCC2a-depleted newborns *in vivo* does not derive from an intrinsic alteration in the central breathing command arising from the two main respiratory oscillators operating in combination.

### Upper airway motor output is unaffected in KCC2a^–/–^ mice

**Figure 5.**
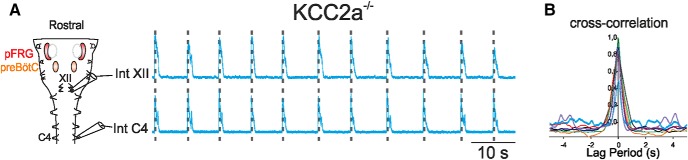
Hypoglossus motor output is normal in KCC2a^–/–^ mutants at P0. ***A***, Left: Schematic representation of an isolated medullary preparation containing both the pFRG (red shading) and preBötC (orange shading) networks with macroelectrodes placed on ventral rootlets to record rhythmic respiratory activity from the phrenic (C4) and hypoglossal (XII) nerves simultaneously. Right: Synchronized activity (indicated by dashed vertical lines) seen in integrated traces recorded from C4 (Int C4, bottom trace) and hypoglossal (Int XII, top trace) nerves in a KCC2a^–/–^ preparation at P0. ***B***, Cross-correlation histogram showing perfect 1:1 synchronized bursting in the C4 and the XII rootlets of 7 different mutant preparations. The complete absence of any skipped cycles at the level of the hypoglossal root indicated that apneas observed *in vivo* are not due to a dysfunction of the upper airways motor command.

Thus far, our data have provided strong evidence that the apneas expressed by KCC2a^–/–^ mutants *in vivo* are not due to a major dysfunction of the central command emanating from the rhythmogenic respiratory circuitry. However, apneas can also originate more peripherally due to a malfunction of motor output transmission to the muscles of the upper airways. Because KCC2a is also normally strongly expressed in the hypoglossal nuclei (see Fig. 2*C*) that mediate a component of this motor drive, we assessed whether the mutants’ respiratory pauses *in vivo* resulted from deficits in the production of hypoglossal motor output. In whole isolated medullary preparations obtained from KCC2a^–/–^ newborns at P0, we simultaneously recorded the C4 and XII roots that carry axons of two major motoneuronal pools (phrenic and hypoglossal) driven by the preBötC network and that control respiration-related muscles of the diaphragm and upper airways, respectively. As illustrated in Fig. 5*A*, rhythmic coactive inspiratory bursts were systematically observed in the phrenic and hypoglossal roots of all mutant preparations tested (*n* = 7). No sign of any skipped cycles was detected in the hypoglossal activity over recording periods lasting up to 15 min, as confirmed by cross-correlation analysis (Fig. 5*B*), which showed a strict 1:1 synchrony in the bursting activity of the two roots. Thus, an anomaly in the motor output to the upper airways was not detectable in our isolated medullary preparations that would have otherwise indicated a failure in transmission of the central respiratory drive to hypoglossal motoneurons.

It should be noted, furthermore, that these *in vitro* findings were consistent with visual inspection of rib cage displacements during actual breathing expressed by the freely-behaving newborn mutant, where periods with a complete absence of respiration-related movement, including contraction of the diaphragm and intercostal muscles, were observed (see Video 2). Such intermittent pauses were never observed in wild-type control animals (see Video 1). This observation thus further indicated that the mutant’s apneas *in vivo* were not due to an obstruction of the upper airways, but rather, arose from a transient perturbation of global central respiratory circuit operation, possibly caused by abnormal sensory feedback inputs (that are only present *in vivo*) or abnormal central modulatory influences.

**Video 1.** Ventral view of P0 newborn KCC2a^+/+^ (1) and KCC2a^–/–^ (2) animals. Note the prolonged and recurrent apneas expressed by the mutant.

**Video 2.** Ventral view of P0 newborn KCC2a^+/+^ (1) and KCC2a^–/–^ (2) animals. Note the prolonged and recurrent apneas expressed by the mutant.

### Enhancement of inhibitory pontine influences on central respiratory circuits in KCC2a^–/–^ mice

**Figure 6.**
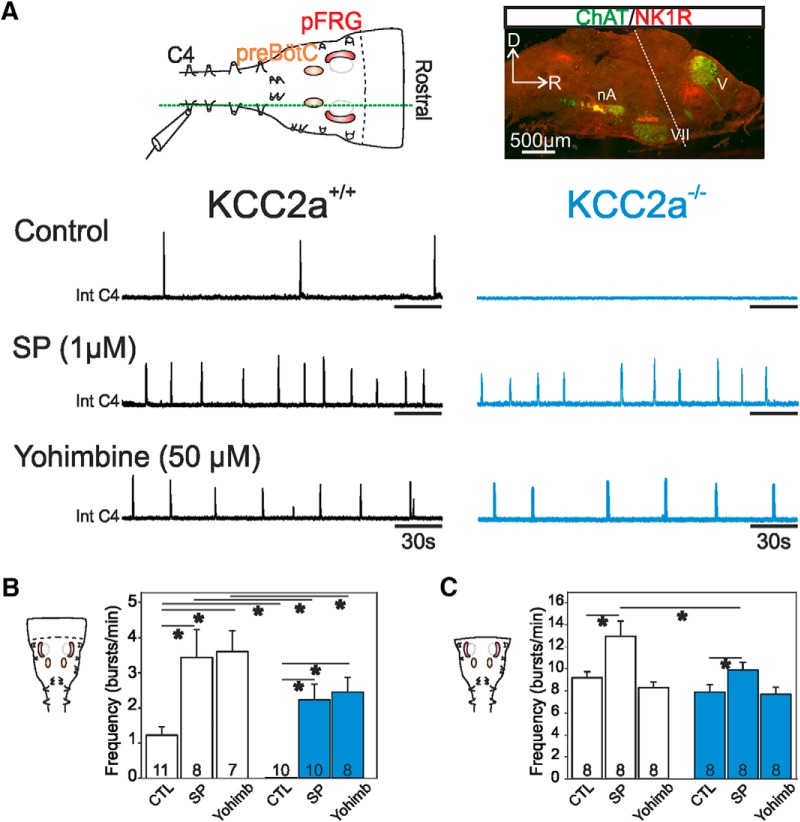
The extrinsic inhibitory influence from the pons is stronger in KCC2a^–/–^ mutants at P0. ***A***, Top left: Schematic representation of an *in vitro* pontomedullary preparation containing the pontine structures and the pFRG (red shading) and preBötC (orange shading) networks with a macroelectrode placed on a phrenic rootlet (C4) to record rhythmic respiratory activity. The dashed green line indicates the level of sectioning for the image at right. Top right: Sagittal slice of a pontomedullary preparation showing the position of motoneuronal groups (ChaT immunoreactive populations in green) such as in the trigeminal nucleus (V), the facial nucleus (VII), and the nucleus ambiguus (nA). The black dashed line indicates the border between the pons and the medulla (also corresponding to the anterior boundary of isolated medullary preparations as shown in Fig. 3). Lower panel: Integrated traces of C4 activity (Int C4) in control conditions (top traces), in the presence of 1 µM SP (middle traces) and after application of the α2-AR antagonist yohimbine (50 µM; bottom traces) in KCC2a^+/+^ (black traces at left) and KCC2a^–/–^ (blue traces at right) pontomedullary preparations at P0. Respiratory activity was completely absent in the mutant in control conditions but could be triggered by application of the excitatory neuromodulator SP or the α2-AR antagonist yohimbine (50 µM). ***B***, Histograms depicting mean respiratory cycle frequency values (± SEM) obtained in KCC2a^+/+^ (unfilled bars) and KCC2a^–/–^ (blue bars) pontomedullary preparations in control conditions, in the presence of 1 µM SP and in the presence of 50 µM yohimbine. ***C***, Same layout as in ***B*** for measurements obtained from isolated medullary preparations (i.e., without the pons). Asterisks indicate statistical differences (*p* < 0.001). The number of experiments in each condition is indicated in the corresponding column.

Given that the respiratory networks of KCC2a mutants remain intrinsically capable of generating regular rhythmic activity when isolated, yet produce a slower rhythm with recurrent episodes of apnea *in vivo,* one remaining explanation is that extrinsic neuromodulatory influences, notably those exerted by the pons, are affected by the mutation and thus perturb downstream network function. To test this possibility, we recorded respiration-related activity in brainstem *in vitro* preparations that additionally included the pons (Fig. 6*A*). The major observable difference between such pontomedullary preparations from KCC2a^+/+^ and KCC2a^–/–^ newborns was that spontaneous rhythmic activity was completely absent in the C4 root of mutant preparations (*n* = 10), whereas all wild-type preparations (*n* = 11) continued to generate a respiratory rhythm, albeit at a lower mean frequency (1.22 ± 0.2 bursts/min) than when the pons was absent (Fig. 6*A*, *B*). This finding therefore suggested that the presence of the pons maintains the respiratory networks in a much less excitable state in the mutant than in the wild type. To further test this idea, we bath-applied different pharmacological agents to modify the modulatory environment to which the respiratory rhythm-generating networks were exposed. First, exogenous application of SP (1 µM), a known excitatory modulator of respiratory circuit activity (Gray et al., 1999; Wang et al., 2001), induced an increase in the ongoing rhythm frequency in wild-type preparations to a mean value of 3.43 ± 0.7 bursts/min (Fig. 6*A*, left; *B*). In mutant pontomedullary preparations that were otherwise silent under normal aCSF, SP triggered onset of rhythmic activity at a frequency of 2.22 ± 0.4 bursts/min (Fig. 6*A*, right; *B*), thus indicating that the respiratory networks were indeed held depressed under normal saline conditions, yet were still potentially functional.

A second bath-application experiment concerned the A5 neuronal group located in the pons, which is known normally to inhibit the respiratory rhythm via the activation of α2 adrenergic receptors (α2-AR; Jodkowski et al., 1997; Dawid-Milner et al., 2001; Hilaire et al., 2004; Viemari, 2008). To test the effective influence of this catecholaminergic modulation on spontaneous respiratory network activity, we then applied yohimbine (50 µM), an α2-AR antagonist, to our pontomedullary preparations. In wild-type preparations, blockade of α2-AR induced a significant increase in respiratory frequency to 3.59 ± 0.5 bursts/min (Fig. 6*A*, left, *B*), whereas in mutants, the cycle rate increased to a mean of 2.45 ± 0.4 bursts/min (Fig. 6*A*, right, *B*). That the rhythm frequencies expressed by preparations of the two genotypes in the presence of SP or yohimbine were both statistically different (*p* = 0.03) further supported the conclusion that the respiratory rhythm in the mutants was subjected to a stronger depressive modulatory influence than in wild-type preparations. It should be noted that the A1/C1 and A2/C2 noradrenergic groups located in the medulla also exert modulatory effects on the respiratory networks via α2-AR activation. But since these influences can be either facilitating or stabilizing (Viemari, 2008), they do not readily comply with the different antagonist effects observed here. Furthermore, the more modest changes observed in mutant preparations after blockade of α2-AR could also be indicative of inhibitory influences deriving from other pontine structures.

To further test the hypothesis that the pontine circuits are indeed responsible for the breathing anomalies in the mutant, we performed similar pharmacological applications to isolated medullary preparations, which thus was in the absence of the pons. Exogenous application of 1µM SP induced an increase in respiratory rhythm frequency in wild-type preparations (*n* = 8) from a control mean value of 9.1 ± 0.5 bursts/min to 12.9 ± 1.3 bursts/min (*p* = 0.02; Fig. 6*C*). In contrast, application of 50 µM yohimbine to the wild-type brainstems had no significant effect on the ongoing rhythm frequency (8.2 ± 0.5 bursts/min; *p* = 0.2). In mutant medullary preparations (*n* = 8), SP still induced an increase in respiratory activity from a mean frequency of 7.9 ± 0.6 bursts/min in control conditions to 9.8 ± 0.7 bursts/min (*p* = 0.05) under SP (Fig. 6*C*). Significantly, on the other hand, yohimbine treatment remained without any significant effect on the control mean frequency of 7.6 ± 0.6 bursts/min, *p* = 0.7 (Fig. 6*C*). Taken together, therefore, these latter findings indicate that SP directly modulates neuronal groups, including the two main respiratory networks, contained within the brainstem of both wild-type and mutant preparations. In contrast, the effect of α2-AR blockade on rhythmogenesis seen in pontomedullary preparations (but not in medullary preparations) originates mainly in the pons itself. Furthermore, a stronger pontine inhibitory modulation, at least partly exerted by the A5 group through the activation of α2-AR, offers an explanation for the slower breathing frequency observed in the mutant *in vivo* and may also facilitate the expression of apneas by reducing respiratory network excitability.

**Figure 7.**
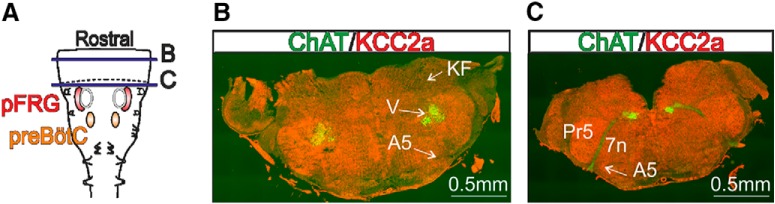
The KCC2a cotransporter isoform is also expressed in the pons. ***A***, Schematic of a pontomedullary preparation including the pFRG and preBötC respiratory networks. The black dashed line indicates the boundary between the pons and the medulla. ***B***, ***C***, Immunostainings against KCC2a (red) and ChAT (green) at the two different transverse levels indicated by solid horizontal violet lines in ***A*** in a KCC2a^+/+^ preparation at P0. KCC2a is widely expressed throughout the pons. A5, adrenergic group 5; KF, Kölliker-Fuse; Pr5, trigeminal sensory nucleus; V, trigeminal motor nucleus; 7n, seventh nerve.

A prerequisite for the above hypothesis is that KCC2a is normally present in the pons of newborn animals. As for other medullary structures (see Fig. 2), we therefore performed immunostaining directed against KCC2a (Uvarov et al., 2009; Markkanen et al., 2014) in the pons of wild-type preparations (Fig. 7*A*) in conjunction with ChAT antibody labeling to locate pontine motoneuronal groups, and more specifically the trigeminal motor group and the facial nerve, to provide anatomic landmarks. As illustrated in the more rostral (*B*) and caudal (*C*) sections of Fig. 7, KCC2a is widely expressed in almost all pontine tissue, including the Kölliker-Fuse and the A5 and A7 adrenergic groups. This expression pattern is thus consistent with the idea that perturbing KCC2a is likely to have functional consequences for pontine neuronal groups that send suppressive inputs to downstream medullary rhythmogenic networks.

## Discussion

In this study, we report that the neuronal-specific KCC2 chloride cotransporter is involved in breathing activity at the time of birth. This is at least partly due to a contribution of the KCC2a isoform that we found to be expressed at birth in brainstem regions where respiratory neuronal groups are located and the absence of which induces quantifiable breathing anomalies *in vivo*. Specifically, in newborn pups deficient for KCC2a, we observed a significantly slower breathing frequency than in wild-type animals, in conjunction with an elevated occurrence of apneas. However, in *in vitro* preparations that isolate one or both of the two major respiratory oscillators (the preBötC and the parafacial respiratory group), rhythmic respiratory-related activity is generated at a normal frequency with no evidence of recurrent fictive apneas. In addition, the downstream motor command for the upper airways emanating from the hypoglossus nucleus appears to be fully operational in the mutant. Altogether, these results indicate that the reduction in rhythm frequency and the generation of apneas in the absence of KCC2a are not a direct consequence of respiratory oscillator network dysfunction per se. Rather, a transient abnormal enhancement of pontine inhibitory neuromodulatory influences arising upstream from the medullary respiratory networks could maintain the latter in a more depressed state in the mutant, thereby resulting in a reduced breathing frequency and facilitating the genesis of apneas. On this basis, therefore, our data show for the first time that the KCC2a isoform does indeed play an important, albeit indirect, role in ensuring the correct expression of respiratory rhythmogenesis at the time of birth.

In attempting to determine the central sites at which the breathing anomalies observed *in vivo* in the KCC2a mutants originate, we performed several sets of complementary experiments using different *in vitro* preparations. Clearly, each of these reduced preparations (transverse medullary slices, isolated medullary preparations, pontomedullary preparations) represents an incomplete part of the whole respiratory system and therefore care must be taken in translating mechanisms of neural circuit function established *in vitro* to their role in the intact animal. Additionally, the employment of a significantly higher extracellular potassium concentration in the aCSF (which is commonly used in *in vitro* studies on the respiratory networks), which allows the generation of stable rhythms *in vitro* for several hours, is not strictly comparable to the *in vivo* condition. Despite these caveats, *in vitro* studies have been extremely useful in gaining insights into the origins and operation of the respiratory central command (for example, Smith et al., 1991; Feldman and Del Negro, 2006; Garcia et al., 2011; Ramirez et al., 2012; Feldman et al., 2013), and it is therefore reasonable to predict that our *in vitro* findings on the respiratory phenotype of the KCC2a mutants reveal at least some of the neural processes occurring *in vivo*.

The first noticeable breathing deficit of KCC2a mutants *in vivo* was an abnormally low respiratory rhythm frequency. Our immediate assumption was that this slowing was due to a functional defect in the central neuronal networks that generate and control the respiratory rhythm, namely the preBötC and the pFRG. This hypothesis was initially supported by the confirmation that the KCC2a isoform is strongly expressed in the brainstem regions encompassing the preBötC and the pFRG of wild-type animals. Unexpectedly, however, when isolated *in vitro*, the preBötC network appeared to function normally in the mutant lacking KCC2a, suggesting this isoform is not after all involved, at least in regulating inspiratory rhythmogenesis. One possibility was that the remaining KCC2b isoform compensated for the lack of KCC2a in a manner sufficient to sustain the generation of respiratory activity (also see below). Such a substitutive process has been proposed to occur in other regions of the neonatal mouse brainstem where the two isoforms are codistributed (Markkanen et al., 2014). Although we also found KCC2b normally to be colocalized with KCC2a in medullary respiratory regions, our present data do not allow us to assess this possibility.

A further plausible explanation for the slower frequency in the mutant is a deficiency in the preBötC’s partner pFRG oscillator or in the synaptic coupling between the two networks (Mellen et al., 2003; Feldman and Del Negro, 2006; Thoby-Brisson et al., 2009; Bochorishvili et al., 2012). Indeed, it is known that the interconnected pFRG and preBötC circuits influence each other’s frequency and that the absence of a functional pFRG results in a slower preBötC rhythm frequency recorded from isolated hindbrain preparations (Pagliardini et al., 2008; Dubreuil et al., 2009; Rose et al., 2009; Thoby-Brisson et al., 2009; Caubit et al., 2010). However, here again we found no difference in the fictive respiratory activity recorded from *in vitro* preparations containing both networks obtained from KCC2a mutant animals compared to wild-type preparations. Based on these findings, therefore, it can be concluded that an intrinsic dysfunction of neither the pre-Bötzinger or pFRG networks, nor their synaptic interactions, is the source of the slower breathing frequency observed *in vivo* in KCC2a^–/–^ newborn animals.

The central command for breathing is also under the influence of neuromodulatory inputs arising in different parts of the CNS, including more proximal nuclei of the pons (Doi and Ramirez, 2008; Dutschmann and Dick, 2012). The overall influence of the pons on the respiratory rhythm is inhibitory, with the latter being generated at a lower frequency in isolated preparations in which the pons is left attached to the medulla. Among the pontine neuronal systems that modulate breathing, the A5 adrenergic group is known to exert strong inhibitory effects on the respiratory command through the activation of α2-AR (Viemari, 2008), although probably indirectly, since the presence of α2-AR on inspiratory pre-Bötzinger neurons has yet to be established (Viemari and Ramirez, 2006). Nevertheless, a reasonable further explanation for the depressed breathing behavior in the KCC2a mutant is that the activity of the A5 group becomes stronger and thereby more effectively inhibits the activity of its respiratory network targets. In experimental support of this idea, antagonizing α2-AR in pontomedullary preparations from the mutant appears to disinhibit the respiratory networks and trigger the generation of rhythmic activity that was previously totally absent.

However, it remains unclear as to how the deletion of one KCC2 isoform leads to the strengthened inhibitory influence of the A5 group on respiratory rhythmogenesis. Here again, one possibility is that the KCC2b isoform compensates for the absence of KCC2a. Because KCC2b is associated with a more mature chloride-mediated signaling (Markkanen et al., 2014; Uvarov et al., 2009), A5’s inhibitory influence might effectively become established earlier in KCC2a^–/–^ mutants than would otherwise occur during normal postnatal development. Such a compensatory mechanism could in turn lead to the respiratory rhythmogenic networks being already subjected to the stronger suppressive influence of pontine neuromodulatory structures at birth, resulting in the generation of breathing activity at an even lower frequency and a greater susceptibility to the expression of apneas. However, it is likely that the respiratory activity phenotype found in the KCC2a^–/–^ mutant is the consequence of changes at multiple sites.

The second major breathing anomaly of KCC2a mutants at P0 was the recurrent occurrence of apneas. However, these respiratory pauses were detected *in vivo* but not in reduced *in vitro* medullary preparations, suggesting that they too were not of a central respiratory network origin. Another form of respiratory pausing, termed obstructive apnea, is primarily unrelated to a cessation in the central respiratory command itself, but rather, is caused by a collapse or occlusion of the pharyngeal airway that leads to an increased air passage resistance and an interruption of air inflow (Eckert et al., 2009; Ramirez et al., 2013). Of relevance in this context is our finding that KCC2a in wild-type mice at P0 is strongly expressed in hypoglossal motoneurons, which innervate the genioglossus muscle that pulls the tongue forward to enlarge the upper airways during each inspiratory phase of breathing. Failure of such phasic genioglossus muscle contractions driven by hypoglossal motoneurons induces pharyngeal collapse that ultimately may result in a complete upper airway blockade and the expression of obstructive apnea (Ramirez et al., 2013). It is possible, therefore, that a deficiency in hypoglossal motoneuron output was responsible for the frequent occurrence of apneas that we observed in the KCC2-deficient mouse pups *in vivo*. However, our *in vitro* data showed that when isolated from upstream pontine inputs, the central respiratory drive from preBötC interneurons and premotoneurons to hypoglossal motoneurons remained fully efficient in the mutant. This in turn suggests that failure in the transmission of inputs from the rhythmogenic network to the motoneurons cannot be the direct cause of apneas *in vivo*. Interestingly, the hypoglossus nucleus contributes to genioglossus muscle contractions through many types of both phasic and tonic motor units (Hwang et al., 1983; Haxhiu et al., 1992; Saboisky et al., 2007). Therefore, it remains possible that a deficit in tonic hypoglossus activity might also contribute to a transient collapse of upper airway muscle contractions, which additionally may arise peripherally from a dysfunction at the level of the neuromuscular junction. In any case, the mechanisms leading to apneas are still not fully understood, and it is very likely that multiple underlying factors and events are involved.

Indeed, a further explanation relates to other inputs to hypoglossal motoneurons, including influences from the Kölliker-Fuse nucleus (KF; Bautista and Dutschmann, 2014; Silva et al., 2016; Yokota et al., 2016). The KF, which is located in the dorsolateral pons, is an important center for respiratory rhythm control and the regulation of upper airways patency. This nucleus sends projections to several brainstem areas involved in breathing, including the retrotrapezoid nucleus, the ventral respiratory group, the phrenic nucleus, and the hypoglossus nucleus (Dutschmann and Dick, 2012). Thus, disturbances to KF neuromodulatory influences on respiratory motoneurons in the KCC2a^–/–^ mutant could underlie the respiratory pauses occurring *in vivo*. Furthermore, the hypoglossal motoneurons also receive inputs from noradrenergic neurons from the A6 and A7 groups (Aldes et al., 1992), so here again, anomalies in these excitatory inputs could be another source of breathing deficiency. Because respiratory apneas generally result from complex interactions between both peripheral and central nervous system factors (Eckert et al., 2009; Ramirez et al., 2013), it is probable that the respiratory pauses occurring *in vivo* in association with KCC2a deletion are not attributable to a single cause. In further support of this conclusion, the visual inspection of respiratory behavior by KCC2a mutants revealed a complete absence of breathing movement, including diaphragm displacement, during each apnea, indicating a cessation of activity by the entire (both central and peripheral) respiratory apparatus.

The phenotype of respiratory activity observed in KCC2a mutants is transient, as it is mostly evident at P0, before a regular, albeit still slower, rhythm emerges over the ensuing days. It is noteworthy that the respiratory phenotype of the KCC2b-deficient newborn has thus far not been described in detail in the literature (Woo et al., 2002; Uvarov et al., 2007), other than the generation of atypical sigh-related activity observed *in vitro* in the mutant at embryonic stages (Chapuis et al., 2014). Although both the KCC2a and KCC2b isoforms are present in brainstem structures at birth, KCC2a represents only 20% to 50% of the total amount of KCC2 mRNA in the neonatal mouse brainstem (Uvarov et al., 2007). In addition, the expression of KCC2b increases postnatally to become widely expressed in the adult CNS (Uvarov et al., 2007, 2009; Markkanen et al., 2014). Therefore, even if KCC2b is able to compensate for the lack of KCC2a in the mutant at birth, in any case the former isoform becomes functionally predominant during the subsequent postnatal period, and accordingly, the consequences of a KCC2a mutation will become progressively less important. It is presumably for this reason that by the time they reach adulthood, KCC2a-deficient mice no longer exhibit any obvious behavioral phenotype related to the mutation (Markkanen et al., 2014).

A further developmental aspect to consider is the significant changes that occur in chloride-mediated signaling. Specifically, expression of the chloride cotransporters NKCC1 and KCC2 is developmentally regulated, with NKCC being predominant at early (including embryonic) immature stages where, by maintaining high [CL^–^]_I_, it produces a depolarizing action of chloride-mediated signaling. Chloride-extruding KCC2 then becomes dominant during later development to promote the hyperpolarizing effect of chloride-mediated transmission (Rivera et al., 1999; Ben-Ari, 2002). Consistent with this transition, it has been shown that during the first two postnatal weeks, NKCC1 expression in respiratory-related nuclei of the brainstem (including the pFRG and preBötC) undergoes a developmental decrease, while concomitantly, KCC2 expression increases (Liu and Wong-Riley, 2012; Okabe et al., 2015). Neuronal chloride concentrations also change in brainstem respiratory circuits of rodents during the perinatal period (Ren and Greer, 2006), further pointing to the requirement for the proper expression of the different KCC2 isoforms at different developmental stages. Accordingly, when both KCC2 isoforms are genetically deleted, newborn animals exhibit severe motor deficits that result in a complete lack of breathing behavior (Hübner et al., 2001), thus indicating that KCC2 cotransporters are critically important for the proper establishment of an efficient respiratory function. Because synaptic inhibition is essential for generating stable and properly organized respiratory motor patterns and movements by coordinating the inspiratory/expiratory phases of neuronal activity within and outside the central rhythmogenic networks, any disturbances in chloride-mediated signaling would have major consequences for breathing behavior.

## Synthesis

Reviewing Editor: Douglas Bayliss, University of Virginia School of Medicine Decisions are customarily a result of the Reviewing Editor and the peer reviewers coming together and discussing their recommendations until a consensus is reached. When revisions are invited, a fact-based synthesis statement explaining their decision and outlining what is needed to prepare a revision will be listed below. The following reviewer(s) agreed to reveal their identity: Jeffrey Smith, Mathias Dutschmann.

This manuscript was seen by two reviewers, who agree that this is a technically sound piece of work that provides new information on a transient contribution of the Kcc2a isoform in respiratory circuit function. As you will see from their comments, they have each provided a number of helpful suggestions for improvement, including points where further discussion and clarification are required. For example, it will be important to discuss further the caveats associated with extrapolating results from the neonatal in vitro preparations to mechanisms underlying respiratory behaviors in vivo; also, please provide more detail on antibody validation and characterization.

Of particular note, the reviewers concur that interpretations regarding the involvement of descending modulatory inputs from the pons (A5 group) would be strengthened by experiments using yohimbine (or other α2-AR antagonists) in more reduced in vitro preparations (slice and medulla-spinal cord preparations). It was suggested that further elaboration of experimental evidence from Viemari, 2008 could potentially satisfy this concern.

Please find below the full reviews.

Reviewer #1

Advances the Field

Genetic deletion of neuronal-specific potassium-chloride co-transporters KCC2, which are critical for maintaining an appropriate chloride gradient for chloride-mediated synaptic inhibition, completely disrupts breathing behavior in newborn mice. It is therefore important to understand the roles of individual KCC2 isoforms (2a and 2b), which have different developmental expression profiles, in development of normal respiratory motor function postnatally. This novel study examines disturbances of respiratory activity in neonatal mutant mice lacking the KCC2a isoform, which is expressed at birth, but whose role in the proper operation of brainstem respiratory circuits has not been defined. The authors provide novel evidence for expression of the KCC2a isoform in brainstem respiratory-related regions and a transient role of KCC2a in the generation of stable respiratory activity and circuit function at birth from for the most part technically sound in vivo and in vitro experimental analyses.

Multimedia Comments

The two movies included are useful but not necessary.

Comments to the Authors

This study examines disturbances of respiratory activity in neonatal mutant mice lacking the neuronal-specific potassium-chloride co-transporter KCC2a isoform, which is expressed at birth, but whose role in brainstem respiratory circuits is unknown. In general, genetic deletion of KCC2 co-transporters, which are critical for maintaining an appropriate chloride gradient for chloride-mediated synaptic inhibition, completely disrupts breathing behavior in newborn mice. It is therefore important to understand the roles of individual KCC2 isoforms (2a and 2b), which have different developmental expression profiles, in development of normal respiratory motor function postnatally. From plethysmographic measurements of neonatal mutant mouse breathing behavior in vivo, the authors provide novel evidence for a transient role of the KCC2a isoform in the generation of stable breathing and respiratory circuit function. Specifically, the data presented indicate that newborn KCC2a-deficient mice exhibit a slower breathing frequency and a higher incidence of apneas over the first several postnatal days compared to the control wild-type mice.

Importantly, the authors test for anomalous function of brainstem respiratory circuits, focusing on circuit activity at P0 when the breathing disturbances were most prominent in vivo, by analyzing respiratory activity from isolated in vitro neonatal slice as well as en bloc medullary-spinal cord or intact brainstem-spinal cord preparations capturing various circuit components. Expression of the KCC2a isoform was also analyzed and verified by immunolabeling in brainstem respiratory-related regions including the preBötC region, hypoglossal motor nucleus, pFRG/RTN region, and several pontine regions including the Kölliker-Fuse nucleus and A5 group. The measurements of inspiratory activity from isolated in vitro preparations (preBötC activity in slices, hypoglossal and phrenic activity in en bloc medullary-spinal cord preparations) indicate normal inspiratory rhythm generation and motor output patterns in the mutants compared to control preparations. Measurements of respiratory activity as well as pharmacological tests in the en bloc brainstem-spinal cord preparations including the pons suggest a stronger pontine inhibitory modulation of downstream medullary circuits in the mutant neonatal mice, at possibly least in part by the pontine A5 group through activation of α2-adrenergic receptors, as a possible explanation for the slower breathing frequency and reduced network excitability leading to expression of apneas in the mutant in vivo. In general, the authors infer that perturbing KCC2a has transient functional consequences for pontine neuronal groups that provide inputs to downstream medullary rhythmogenic circuits, although it remains unclear how the deletion of one KCC2 isoform specifically leads to augmented inhibitory modulation of inspiratory rhythm by the A5 group.

Overall the experimental strategy is reasonably well thought out and technically sound although some additional technical details are required. The Discussion of the results and issues related to the developmental role of KCC isoforms in respiratory circuit function is insightful, including considering that the very transient nature of the breathing disturbances in the KCC2a-deficient mutants in vivo may be attributable to the remaining KCC2b isoform compensating sufficiently to stabilize generation of respiratory activity postnatally. Although analyzing respiratory circuit function in the in vitro preparations is a reasonable strategy, there are caveats associated with this approach, since it is not known precisely how mechanisms (e.g., for rhythm generation and regulating circuit excitability) operating in vitro translate to circuit function in vivo, which needs to be noted in the Discussion. The general concept that KCC2 co-transporters are critical for proper establishment of normal respiratory circuit function is important to highlight.

Suggestions for revision.

Introduction

p. 3, para. 1, lines 45-46. “...whereas inhibitory synaptic transmission, although not critical for rhythmogenesis per se..." I suggest deleting “although not critical for rhythmogenesis per se”, since involvement of synaptic inhibition is thought to be state-dependent. For example, it is established that inspiratory rhythm generation occurs in the reduced neonatal in vitro preparations without critical involvement of synaptic inhibition, but in more intact preparations including in vivo, some studies in the reference list cited here indicate that there are major disturbances of the rhythm along with the disturbances of respiratory pattern with disruption of synaptic inhibition. Also, the authors note that complete disruption of breathing occurs in neonatal transgenic mice without the two KCC2 isoforms suggesting an important functional role developmentally.

p. 4, para. 1, lines 82-83. “Our data indicate that KCC2a's regulatory role is accomplished upstream from the central rhythm-generating networks...” Better to state: “...may be accomplished by pontine mechanisms upstream from the medullary rhythm-generating networks...”

Methods

p. 5, para. 3. Provide more details of the plethysmography system used- e.g., was this a flow-through (bias flow) chamber?

p. 6, para. 1, line 123. “ ...continuously bubbled with oxygen.” Bubbled with 95% O2-5% CO2? Also, in line 141, indicate that the preparations were maintained in the same aCSF.

p. 7, para. 3 (Immunostaining). State how the antibodies were validated. Antibodies for KCC2a were presumably validated by the absence of immunolabeling in the KCC2a isoform (Fig. 3C). If the antibody for KCC2b was previously validated using a KCC2b null mutant, this should be referenced.

Results

p. 9, para. 1. The authors show relatively low-magnification immunostaining for the KCC2a isoform, and it would be useful to also show some higher cellular resolution images of the staining localized to neuronal membranes. The diffuse nature of the immunostaining shown implies labeling of inhibitory neuron terminals, and it would be useful to document in more detail the labeling localization, or at least the authors should provide information in the text in this regard.

p. 9, para. 1, lines 238-240. “...at a more caudal level where closure of the fourth ventricule (correct spelling here) is starting, a strong KCC2a staining was found in the region where the hypoglossal nuclei are located (Fig. 2C).” The hypoglossal motor nucleus extends more rostral than the level described here and many respiratory XII motoneurons are located at these more rostral levels. The authors should point out that there was also immunolabeling within the XII motor nucleus more rostrally (Fig. 2B). The strong immunolabeling within the hypoglossal and other motor nuclei implies expression of KCC2a within inhibitory terminals innervating the motoneurons, which should be mentioned.

p. 11, para. 2, lines 312-313. “...KCC2a is also normally strongly expressed in the hypoglossal nuclei (see Fig. 2) that mediate this motor drive” Insert “a component of” after “mediate”.

p. 13, para. 2, line 391. “...consistent with the idea that perturbing KCC2a signaling...” Remove “signaling”.

Discussion

p. 16, para. 3, line 504. “retro-trapezoid”. Remove the hyphen.

p. 17, para. 2. The authors should mention what is known about the respiratory phenotype in KCC2b-deficient mice that die at three-weeks postnatally.

Figures and Legends

Figure 2. Indicate the thickness (30 µm) of the histological sections in the legend. In panels 2B, the area outlined (right panel) for the preBötC region extends too medially, although it is clear that in the region, more directly ventral to the nA that typically would correspond to the preBötC region, there is also KCC2a immunolabeling. This also applies to Fig. 3C right panel. Also, the slice shown in 2B appears to be cut at an angle such the ventral half of the slice is at a more rostral level than the dorsal part, and accordingly the ventral region may actually be somewhat rostral to the typical level of the preBötC. This should be checked.

Figure 3. In Fig. 3C the authors show the absence of immunostaining in the KCC2a null mutant, which is important for antibody validation, although the image shown indicates substantial background labeling with their antibody and the immunolabeling procedures used, but clearly less labeling than in the wild-type controls. It is worth mentioning in the legend that the labeling shown is background in the null mutant, which is substantially below the labeling in the tissue from wild-type animals.

Figure 4 legend, line 744. Suggest replacing “macroelectrode” by “suction electrode”. Lines 745-746. “The dashed green line indicates the sectioning level...” The sectioning level indicated does not pass through the preBötC region in the schematic, which is inconsistent with the actual parasagittal section shown. Lines 753-754. “...together with mean values for individual preparations”. Indicate the total number of cycles or duration of the period used to obtain the mean values.

Reviewer #2

Advances the Field

First investigation of the role KCC2a in neonatal respiratory control.

Comments to the Authors

This manuscript is addressing the role of KCC2a in neonatal respiratory control. The topic is important and interesting. However, I have major points that need to be addressed.

Major:

The dilemma starts with the first sentences of the introduction excitatory neurotransmission is import for respiratory rhythm generation while inhibitory neurotransmission is linked to the formation of the respiratory motor pattern. All techniques to investigate the phenotype of KCC2a knock out mice are focused on respiratory rhythm generation and thus may not reveal the phenotypes linked to pattern formation. I fully agree with this statement and I am aware that pattern formation in newborn preparation is very challenging to address. It would require the implementation of the perfused brainstem preparation of P0 mice which is was never attempted in the literature. However, neonatal (P0-P6) preparations were obtained from rats. Studies on synaptic inhibition show that for instance blocking glycinergic inhibition will shift the postinspiratory activity into inspiration causing paradocxial inspiratory laryngeal adduction. Such a phenotype cannot be detected with the applied techniques but could be at least discussed.

Linking the phenotype partially to the integrity of the pontine A5 group is fine, however, the data would be more conclusive when SP and Yohimbine would be applied in the pre-BotC slice and the pre-BotC pFRG preparation as well. In context of the point above it could be part of your rationale to look into descending pontine inputs that are know to impact on respiratory rhythm generation.

Minor:

Please mention is the background strain for the KCC2a knock out mice? In case it is the B6 mouse strain it should be mentioned that this specific mouse strain is susceptible for central apnea (see works G Hilarie)

How does the high K+ concentration (8mM) of ACSF affect the outcome of the in vitro studies?

Results:

Line 200: please specify the duration of monitoring session.

Lines 215-220 - I think here an analysis of the variability of the respiratory frequency would be more conclusive

Line 351-2 do you mean compared to WT? please correct the sentence

Fig. 2:

NA looks to be located quite lateral? - please comment.

Figure 7

I am aware it is neonatal tissue, but the KF is most likely located more medial in neonates and moves into its final later position with maturation and development of scp (anatomical frameworks for the postnatal maturation of the KF can be found in Dutschmann et al., 2004 Respir Physiol Neurobiol). Currently you place the KF into Pr5 and the KF should be more dorsal and medial..

## Author Response

Dear Dr Bayliss,

We thank you for allowing us the opportunity to submit a revised version of our manuscript for consideration for publication in eNeuro.

We are also grateful to the reviewers for their constructive comments and useful suggestions regarding our work. We have tried to attend to all of these (including the editorial comments) in the revised text and our point-by-point responses are listed below. Please note that all modifications to the revised marked manuscript have been highlighted in blue (Reviewer 1), green (Reviewer 2) or violet for both.

We hope that we have satisfactorily addressed all of the reviewers' concerns and we await your reply in due course.

Yours sincerely (and behalf of the authors),

The authors

Editorial comments (abridged to those requiring author responses):

As you will see from (the reviewers') comments, they have each provided a number of helpful suggestions for improvement, including points where further discussion and clarification are required. For example, it will be important to discuss further the caveats associated with extrapolating results from the neonatal in vitro preparations to mechanisms underlying respiratory behaviors in vivo; also, please provide more detail on antibody validation and characterization.

Please refer to our response to 'Comments to authors' of reviewer 1, and to comment #5 of 'Suggestions for revision' from the same reviewer.

Of particular note, the reviewers concur that interpretations regarding the involvement of descending modulatory inputs from the pons (A5 group) would be strengthened by experiments using yohimbine (or other α2-AR antagonists) in more reduced in vitro preparations (slice and medulla-spinal cord preparations). It was suggested that further elaboration of experimental evidence from Viemari, 2008 could potentially satisfy this concern.

Please refer to our response to major comment #2 of reviewer 2, which involves new experiments as suggested.

Reviewer comments (abridged to those requiring author responses):

Reviewer #1

Comments to authors:

Although analyzing respiratory circuit function in the in vitro preparations is a reasonable strategy, there are caveats associated with this approach, since it is not known precisely how mechanisms (e.g., for rhythm generation and regulating circuit excitability) operating in vitro translate to circuit function in vivo, which needs to be noted in the Discussion.

We fully acknowledge that our in vitro approach based on using variously reduced CNS preparations demands caution in translating data to the in vivo situation. As suggested by the reviewer, this caveat is now pointed out in a new paragraph in the Discussion (p. 15).

Suggestions for revision.

Introduction

p. 3, para. 1, lines 45-46. “...whereas inhibitory synaptic transmission, although not critical for rhythmogenesis per se...” I suggest deleting “although not critical for rhythmogenesis per se”, since involvement of synaptic inhibition is thought to be state-dependent. For example, it is established that inspiratory rhythm generation occurs in the reduced neonatal in vitro preparations without critical involvement of synaptic inhibition, but in more intact preparations including in vivo, some studies in the reference list cited here indicate that there are major disturbances of the rhythm along with the disturbances of respiratory pattern with disruption of synaptic inhibition. Also, the authors note that complete disruption of breathing occurs in neonatal transgenic mice without the two KCC2 isoforms suggesting an important functional role developmentally.

We agree with the reviewer in stating that inhibition does not play a role in actual rhythmogenesis is not fully correct, as shown in several studies that we cite. The sentence has been changed as suggested (p. 4).

p. 4, para. 1, lines 82-83. “Our data indicate that KCC2a's regulatory role is accomplished upstream from the central rhythm-generating networks...” Better to state: “...may be accomplished by pontine mechanisms upstream from the medullary rhythm-generating networks...”

Done as suggested

Methods

p. 5, para. 3. Provide more details of the plethysmography system used- e.g., was this a flow-through (bias flow) chamber?

The plethysmographic system we used was an adaptation of the system supplied by Emka. Due to the size of the animals, the standard Emka chamber is incompatible with the measurement of very small inspired and expired volumes, especially if additionally using a constant flow of fresh air. To circumnavigate this problem, we built a smaller chamber (50mL) in which we placed animals. Since a constant airflow was found to create noise that perturbed the readout of the breathing signal, the animals were positioned in the chamber without providing any airflow. Our recording sessions were short enough to avoid CO2 accumulation in the chamber, indicated by the fact that we never observed any breathing activity changes over time, even for recording sessions lasting 10 minutes.

This is now detailed in the text (p. 6).

p. 6, para. 1, line 123. “ ...continuously bubbled with oxygen.” Bubbled with 95% O2-5% CO2? Also, in line 141, indicate that the preparations were maintained in the same aCSF.

Done as suggested.

p. 7, para. 3 (Immunostaining). State how the antibodies were validated. Antibodies for KCC2a were presumably validated by the absence of immunolabeling in the KCC2a isoform (Fig. 3C). If the antibody for KCC2b was previously validated using a KCC2b null mutant, this should be referenced.

Both the KCC2a and KCC2b antibodies have been validated previously (Uvarov et al., 2009; Markkanen et al. 2014; Markkanen et al., 2017). The specificity of the rabbit anti-KCC2a antibody was validated using brain material from KCC2a knockout mice (Uvarov et al., 2009; Markkanen et al., 2014; Markkanen et al., 2017). The specificity of the chicken anti-KCC2b antibody was validated using brain material from KCC2 knockout embryos (Markkanen et al., 2014). Since the anti-KCC2a and anti- KCC2b antibodies were designed to recognize the unique N-terminal parts of KCC2a and KCC2b isoforms, the chicken anti-KCC2b antibody does not recognize the KCC2a isoform (and vice versa) (Markkanen et al., 2014). The specificity of the KCC2b antibody used in the present study was also further confirmed by comparing with the staining obtained using another KCC2b antibody (kindly provided by Dr. Hubner, Germany). References concerning these antibody validations have been added in the Material and Methods (p. 8).

Results

p. 9, para. 1. The authors show relatively low-magnification immunostaining for the KCC2a isoform, and it would be useful to also show some higher cellular resolution images of the staining localized to neuronal membranes. The diffuse nature of the immunostaining shown implies labeling of inhibitory neuron terminals, and it would be useful to document in more detail the labeling localization, or at least the authors should provide information in the text in this regard.

As reported by Markkanen et al. 2014, the cellular localizations of KCC2a and KCC2b are different. In contrast to KCC2b, KCC2a immunoreactivity is known to be found mainly in distal dendrites and is barely visible in neuronal somata and proximal dendrites (Markkanen et al., 2014; Markkanen et al., 2017), which may explain the relatively diffuse pattern of KCC2a immunostaining. This is now pointed out in the text (p. 10)

p. 9, para. 1, lines 238-240. “...at a more caudal level where closure of the fourth ventricule (correct spelling here) is starting, a strong KCC2a staining was found in the region where the hypoglossal nuclei are located (Fig. 2C).” The hypoglossal motor nucleus extends more rostral than the level described here and many respiratory XII motoneurons are located at these more rostral levels. The authors should point out that there was also immunolabeling within the XII motor nucleus more rostrally (Fig. 2B). The strong immunolabeling within the hypoglossal and other motor nuclei implies expression of KCC2a within inhibitory terminals innervating the motoneurons, which should be mentioned.

The reviewer is correct in pointing out that the hypoglossal nuclei have rostro-caudal extensions that project to brainstem regions beyond that illustrated in our Fig. 2. We have verified that KCC2a staining is indeed present in more rostral slices and this is now noted in the text (p. 10).

However, we do not fully agree with the suggestion that KCC2a immunolabeling should specifically mark inhibitory terminals in the different motor nuclei. The KCC2 chloride co-transporter serves to regulate the intracellular chloride concentration of neurons generally rather than exclusively of inhibitory neurons. Moreover, according to the whole body of literature about KCC2, KCC2 protein is known to be excluded from axons of neurons. Therefore, we don't see how KCC2a expression can be considered solely as localized to inhibitory neurons and their terminals onto motoneurons.

p. 11, para. 2, lines 312-313. “...KCC2a is also normally strongly expressed in the hypoglossal nuclei (see Fig. 2) that mediate this motor drive” Insert “a component of” after “mediate”.

Done as suggested (p. 12).

p. 13, para. 2, line 391. “...consistent with the idea that perturbing KCC2a signaling...” Remove “signaling”.

Done as suggested (p.14).

Discussion

p. 16, para. 3, line 504. “retro-trapezoid”. Remove the hyphen.

Done as suggested (p. 18).

p. 17, para. 2. The authors should mention what is known about the respiratory phenotype in KCC2b-deficient mice that die at three-weeks postnatally.

While we acknowledge the relevance of adding this information, unfortunately we couldn't find any detailed description of the respiratory phenotype of KCC2b deficient mice in the two main studies (Woo et al., 2002; Uvarov et al., 2007) that have so far investigated these mutants. The only relevant data that seems to be available comes from an earlier study showing that a lack of KCC2b causing an abnormal shaping of spontaneous sigh-related events at embryonic stages (Chapuis et al., 2014). This is now noted in the text (p. 18).

Figures and Legends

Figure 2. Indicate the thickness (30 µm) of the histological sections in the legend. In panels 2B, the area outlined (right panel) for the preBötC region extends too medially, although it is clear that in the region, more directly ventral to the nA that typically would correspond to the preBötC region, there is also KCC2a immunolabeling. This also applies to Fig. 3C right panel. Also, the slice shown in 2B appears to be cut at an angle such the ventral half of the slice is at a more rostral level than the dorsal part, and accordingly the ventral region may actually be somewhat rostral to the typical level of the preBötC. This should be checked.

The thickness of the slices used for immunostaining is now indicated in the Fig. 2 legend.

Although the precise anatomical borders of the preBötzinger complex region remain impossible to determine (since a specific marker of this heterogeneous population of neurons is still lacking), we accept that the limits we outlined might have extended too medially. This has been altered accordingly in Fig 2 and Fig 3.

We are unsure as to why the reviewer considers that the slice in Fig. 2b was cut at an angle. The entire series of slices illustrated in Fig. 2 were taken from the same transected brainstem, and we cannot find evidence for any angle cutting of the other slices. Moreover, we were extremely careful to position tissue preparations in the cryostat in a way that the section plane was as coronal as possible. Nevertheless, we verified that a comparable staining was obtained in slices consecutive (i.e., more caudal) to the one chosen for the figure. Thus, we feel confident that the images shown encompassed the region where the preBötC is known to be located, albeit possibly at its more rostral end.

Figure 3. In Fig. 3C the authors show the absence of immunostaining in the KCC2a null mutant, which is important for antibody validation, although the image shown indicates substantial background labeling with their antibody and the immunolabeling procedures used, but clearly less labeling than in the wild-type controls. It is worth mentioning in the legend that the labeling shown is background in the null mutant, which is substantially below the labeling in the tissue from wild-type animals.

We are fully convinced that the very slight staining is indeed background labeling. This is now mentioned in the legend as suggested (p. 24).

Figure 4 legend, line 744. Suggest replacing “macroelectrode” by “suction electrode”. Lines 745-746. “The dashed green line indicates the sectioning level...” The sectioning level indicated does not pass through the preBötC region in the schematic, which is inconsistent with the actual parasagittal section shown. Lines 753-754. “...together with mean values for individual preparations”. Indicate the total number of cycles or duration of the period used to obtain the mean values.

We agree that for rootlet recordings the term suction electrode rather than macroelectrode should be used. We have changed the text accordingly (p. 24).

We have also modified the figure in order to have the indicated sectioning level passing through the preBötC.

The duration (3 min) of the period used to obtain the mean values is now stated in the legend. Note that it was already provided in the Material and Methods section.

Reviewer #2

Major:

The dilemma starts with the first sentences of the introduction excitatory neurotransmission is import for respiratory rhythm generation while inhibitory neurotransmission is linked to the formation of the respiratory motor pattern. All techniques to investigate the phenotype of KCC2a knock out mice are focused on respiratory rhythm generation and thus may not reveal the phenotypes linked to pattern formation. I fully agree with this statement and I am aware that pattern formation in newborn preparation is very challenging to address. It would require the implementation of the perfused brainstem preparation of P0 mice which is was never attempted in the literature. However, neonatal (P0-P6) preparations were obtained from rats. Studies on synaptic inhibition show that for instance blocking glycinergic inhibition will shift the postinspiratory activity into inspiration causing paradocxial inspiratory laryngeal adduction. Such a phenotype cannot be detected with the applied techniques but could be at least discussed.

While these are valid points raised by the reviewer, we believe that they raise issues that are beyond the scope of the present investigation. Our aim here was to assess the overall capability of KCC2 deficient mice to produce respiratory-related activity, rather than assessing how synaptic inhibition contributes to pattern formation versus rhythmogenesis or the extent to which it might be affected in the mutant.

Linking the phenotype partially to the integrity of the pontine A5 group is fine, however, the data would be more conclusive when SP and Yohimbine would be applied in the pre-BotC slice and the pre-BotC pFRG preparation as well. In context of the point above it could be part of your rationale to look into descending pontine inputs that are know to impact on respiratory rhythm generation.

We thank the reviewer for suggesting these further experiments which could support (albeit indirectly) our conclusion that a deregulation of upstream pontine neuronal groups is at least partly responsible for the respiratory phenotype of the KCC2a deficient mouse at P0. To this end, we performed a new series of experiments to test the effect of SP and Yohimbine on isolated brainstem preparations (without the pons) obtained from WT and mutant pups. In these new experiments, whereas the typical modulatory influence of SP was found to be preserved in both phenotypes, Yohimbine application was completely without effect in both WT and mutant preparations. These new findings, which are now reported in a new panel in Fig. 6 and the corresponding text (p. 14), are thus consistent with our hypothesis that pontine circuits are indeed involved in the respiratory phenotype observed in the mutant.

Given that the above findings obtained on brainstem preparations (i.e., with both the pre-BotC and pFRG) are already demonstrative, and since direct inhibitory alpha-2 noradrenergic receptor-mediated effects on inspiratory neurons in medullary slices (i.e., with the pre-BotC alone) are virtually absent, suggesting that A5's modulation of preBotzC circuitry is indirect (Viemari and Ramirez, 2006; Viemari, 2008) we chose not to conduct similar experiments on transverse slices since this would not provide any further pertinent information.

Minor:

Please mention is the background strain for the KCC2a knock out mice? In case it is the B6 mouse strain it should be mentioned that this specific mouse strain is susceptible for central apnea (see works G Hilarie)

The generation of the KCC2a mouse line is detailed in Markkanen et al, 2014. Specifically, heterozygous KCC2a-KO mice were backcrossed into C57BL/6JHsd for three to four generations and intercrossed to obtain KCC2a-KO mice. We are aware of Gerard Hilaire's work showing distinct breathing phenotypes in different mouse lines (Menuet et al., 2011), including the C57Cl/6J strain (Stettner et al., 2008a,b). However, because we used the same C57Bl/6JOlaHsd background lines for wild-type and mutant animals, and because our control animals did not show a high occurrence of apneas, we are convinced that our finding of an abnormally high rate of apneas in the mutant results uniquely from the lack of KCC2a expression, and not from the mouse line itself. The background strain used in our study is now mentioned in the text (p. 6).

How does the high K+ concentration (8mM) of ACSF affect the outcome of the in vitro studies?

This point was also partially raised by reviewer 1 and we agree that we should discuss it a little further in the text. We agree that our in vitro experiments cannot be directly extrapolated to the in vivo situation. The relatively high concentration of potassium in our extracellular bathing solution is one of the modified conditions used to reliably obtain stable respiratory rhythms generated in the different reduced preparations. This condition is commonly used by all labs working on the respiratory networks in vitro, ever since the establishment of the brainstem slice preparation (by Smith et al., 1991) that isolates the preBötC network. Furthermore, in order to be able to compare our data obtained from slice, isolated brainstem or ponto-medullary preparations, we employed the same aCSF composition for all three preparation types investigated. We have added a paragraph in the Discussion to address this point as well as related points raised by reviewer 1 (p. 15).

Results:

Line 200: please specify the duration of monitoring session.

The monitoring session lasted 5 min. This was in fact stated in the original manuscript (now on p. 6 of the revised text).

Lines 215-220 - I think here an analysis of the variability of the respiratory frequency would be more conclusive

Since breathing frequencies were measured during non-apneic periods for both genotypes and because breathing activity was very regular, we feel that an analysis of the variability would not be more informative. Instead we chose to calculate frequency values in breaths per minute as is commonly done and which we believe is more easily interpreted by the reader.

Line 351-2 do you mean compared to WT? please correct the sentence

Indeed we meant compared to wild-type, as stated in the original sentence that was as follows (see revised text p. 13): “This finding therefore suggested that the presence of the pons maintains the respiratory networks in a much less excitable state in the mutant than in the wild-type.”

Fig. 2:

NA looks to be located quite lateral? - please comment.

We find that NA's position in Fig. 2 is no more lateral than in all other preparations that we have examined.

Figure 7

I am aware it is neonatal tissue, but the KF is most likely located more medial in neonates and moves into its final later position with maturation and development of scp (anatomical frameworks for the postnatal maturation of the KF can be found in Dutschmann et al., 2004 Respir Physiol Neurobiol). Currently you place the KF into Pr5 and the KF should be more dorsal and medial..

We accept that the KF may be slightly misplaced in the figure since it has proven difficult to find its exact location in tissue obtained from very young animals in the literature. For this reason, we initially decided to give its position in correspondence with that known in more mature tissue. However, we have now changed the image according to the reviewer's comment.
